# Exposure to outdoor aerospora and associated respiratory health risks among adults in Potchefstroom, North-West province, South Africa

**DOI:** 10.3389/falgy.2025.1568669

**Published:** 2025-04-15

**Authors:** Dorra Gharbi, Frank Harald Neumann, Keneilwe Podile, Marinda McDonald, Jo-hanné Linde, Megan Frampton, Jennifer Leigh Liebenberg, Sarel Cilliers, Tshiamo Mmatladi, Phumelele Nkosi, Keamogestswe Paledi, Stuart Piketh, Jurgens Staats, Roelof P. Burger, Henno Havenga, Rebecca M. Garland, Petra Bester, Pedro Humberto Lebre, Cristian Ricci

**Affiliations:** ^1^ Institute of Plant Sciences, University of Bern, Bern, Switzerland; ^2^ Oeschger Centre for Climate Change Research, University of Bern, Bern, Switzerland; ^3^ Unit for Environmental Sciences and Management, Faculty of Natural and Agricultural Science, North-West University, Potchefstroom, South Africa; ^4^ The Allergy Clinic, Blairgowrie, South Africa; ^5^ Department of Paediatrics & Child Health, Faculty of Health Sciences, School of Clinical Medicine, Rahima Moosa Mother & Child Hospital, University of the Witwatersrand, Johannesburg, South Africa; ^6^ Faculty of Health Sciences, North-West University, Potchefstroom, South Africa; ^7^ Department of Geography, Geoinformatics and Meteorology, University of Pretoria, Pretoria, South Africa; ^8^ Africa Unit for Transdisciplinary Health Research, North-West University, Potchefstroom, South Africa; ^9^ Centre for Microbial Ecology and Genomics, University of Pretoria, Pretoria, South Africa

**Keywords:** aeroallergens, skin prick tests, respiratory diseases, Potchefstroom, pollens, fungi, South Africa

## Abstract

**Background:**

Data on allergic rhinitis and respiratory health metrics are limited for South Africa, with grass pollen as a key outdoor aeroallergen. Exotic trees such as plane trees and ragweed produce highly allergenic pollen, dominating indigenous trees and weeds. Pollen allergy prevalence data is lacking in cities of North-West province such as Potchefstroom.

**Objectives:**

This study aimed to (i) assess the prevalence of allergies to major aeroallergens, including Poaceae (grasses), *Cupressus/Hesperocyparis* (cypresses), *Platanus* (plane tree), *Ulmus* (elm), *Quercus* (oak), *Betula* (birch), *Olea* (olive), *Artemisia* (sagebrush), *Amaranthus* (amaranth), *Plantago* (plantain), *Morus* (mulberry), and *Ambrosia* (ragweed), along with fungal spores such as *Alternaria*, *Cladosporium*, and *Penicillium/Aspergillus*, and (ii) investigate the monthly incidence of major aeroallergens and reactivity levels in sensitized adults in Potchefstroom.

**Methods:**

Skin prick tests (SPTs) were performed on 202 adults aged 18–64 years with confirmed allergic symptoms during a field campaign at North-West University (NWU)'s Potchefstroom campus. A test panel of grass, weed, tree, and fungal spore extracts previously identified via aerobiological monitoring was used. Symptom scores were recorded using ISAAC questionnaires; *Spearman*'s statistical correlation between symptom frequency and monthly aeroallergen concentrations were analyzed.

**Results:**

Among the participants, 184 (91%) exhibited positive SPT reactions: 104 (57%) are monosensitized to pollen, 45 (24%) to fungal spores, and 35 (19%) are polysensitized. Aeroallergen prevalence was higher in females (73%) than in males (27%). The most common pollen allergens were *Cynodon dactylon* (Bermuda grass) (85%), *Zea mays* (maize) (46%), *Platanus* spp. (plane tree) (35%), and *Ulmus campestris* (field elm) (33%). Among fungal spores, *Alternaria* was the most common (93%), followed by *Cladosporium* (27%). A significant and positive statistical correlation was found between allergic rhinitis symptoms and monthly pollen concentrations of *Betula*, *Morus*, *Platanus*, and *Quercus*.

**Discussion & Conclusion:**

This pilot study linked aeroallergens detected in Potchefstroom with allergy profiles of local residents. The findings highlight the need for more comprehensive regional studies that integrate allergen testing with aerobiological data. Raising awareness and implementing health strategies are essential for managing allergic rhinitis in South Africa. More affordable and available SPTs kits, adapted to allergy prevalence in South Africa, are strongly suggested.

## Introduction

The prevalence of allergic conditions is rising in both developed and developing countries (1). Asthma incidence rates are 1%–20%, those of allergic rhinitis range from 1% to 18%, and of skin allergies from 2% to 10% in various populations (2).

Since allergic sensitization patterns differ globally; it is mandatory to understand local sensitization patterns for inhalant allergens for better diagnosis and management of patients. Recent data reported that the prevalence of allergic rhinitis (AR) prevalences in three African countries (Algeria, Egypt, and Nigeria), ranged from 3.6% to 22.8% (1). Overall, limited and inconsistent data on the increase of AR in Africa exist (1). An estimated asthma prevalence rate between 6% and 12%, with national estimates ranging from 2% to 53% is reported for Africa (3).

Research has identified house dust mites (*Dermatophagoides* spp.) as a major factor contributing to the increasing prevalence of allergies for Africa (4). Several studies have reported other causes, such as food allergies and sensitization to biological inhalant allergens such as animal dander (5, 6).

Principal bio-pollutants i.e., pollen grains from anemophilous plants, including trees, grasses, and weeds, as well as fungal spores are dominant outdoor aeroallergens (7). Exposure to these aeroallergens leads to allergic symptoms, ranging from seasonal rhinoconjunctivitis to severe asthma (8).

Most of the research on pollen aerobiology and related health outcomes has been concentrated in the northern hemisphere, particularly in North America and Europe, due to the greater availability of researchers, funding, and advanced infrastructure in these regions (9). In contrast, there is limited data on the incidence of allergies linked to aerospora spectra in Africa, including Southern Africa, and the Global South in general.

From an ecological perspective, unique flora with high biodiversity occurs in nine biomes in southern Africa (10), together with a widespread occurrence of northern hemisphere allergenic plant species introduced as ornamentals, crops or weeds (11). Each biome has a distinct climate and flora and includes, subsequent to indigenous vegetation, alien plants from Europe, North and South America and Australasia (12).

Aerospora diversity is correlated with specific allergy patterns, creating challenges such as (i) the interpolation and extrapolation of aerobiological data from one biome to another (13) and (ii) the determination of potential health outcomes of pollen exposure across South Africa's varied phytogeographical and climatic regions (14).

Climate change presents an additional challenge in integrating and comparing pollen aerobiology measurements across southern African locations to assess current impacts and identify regions at heightened risk (15). Pollen seasons are starting earlier and lasting longer, with higher pollen production, concentration, and allergenicity. Plants are also shifting poleward and upward in mountainous regions (16).

Since 1947, these insights have highlighted early challenges in understanding pollen disorders, or pollinosis, among several South African medical practitioners (17). Ordman (18) communicated that pollinosis in South Africa was predominantly linked to the inhalation of grass pollen, which typically is released during summer. For example, pollen grains of subtropical *Stenotaphrum secundatum* (buffalo grass) are the primary aeroallergens in the Western Cape province of South Africa (19).

A regional study conducted across four South African provinces on 6,775 participants found that Bermuda grass (*Cynodon dactylon*) has the highest frequency of positive SPT reactions, with rates of 31% (Gauteng), 30% (Western Cape), 29% (North-West), and 24% (Eastern Cape) (20). The high occurrence of aeroallergens in different regions of South Africa has been confirmed by the South African Pollen Monitoring Network (SAPNET) (14).

Assessing the health impacts of pollen exposure in different regions of South Africa is crucial (13), especially for the local population and sporting events (21). This includes understanding how environmental conditions affect athlete health and performance in events such as long-distance running, cycling, and triathlons (21).

Since 2022, when pollen monitoring was extended to North-West province, an increasing number of research outputs regarding atmospheric pollen load, seasonality, and related health impacts have occurred (22, 23). Potchefstroom, a city experiencing steady growth, was selected as the ninth pollen monitoring location for SAPNET. The area falls within the temperate Grassland Biome, which is characterized by plains with few trees and shrubs, although patches of savanna exist within city limits (23).

No prior research has investigated the prevalence and characteristics of AR in Potchefstroom. A recent publication highlighted local aerospora diversity and its ecological and allergenic significance (23). In this comprehensive study, a detailed the first qualitative and quantitative analysis of aerial pollen spectra during a 13 months period for Potchefstroom underlines that the dominant pollen types originated from Eurasian and North American neophytic trees such as cypress (*Cupressus sempervirens*, *Hesperocyparis arizonica*), plane tree (*Platanus acerifolia*), pine (*Pinus* spp.), olive (*Olea europaea*), mulberry (*Morus alba*, *Morus nigra*), birch (mostly *Betula pendula*), oak (mostly *Quercus robur*), and ash (predominantly *Fraxinus excelsior*). More importantly, the presence of strongly allergenic pollen produced by the North American invader *Ambrosia* sp. (ragweed) attracted the attention of aerobiologists and allergologists (23, 24).

Recently, a multidisciplinary team of aerobiologists, air quality scientists, clinicians, public health experts, and biostatisticians designed a research project to link aerobiological data to allergenicity, addressing knowledge gaps related to pollen rain and its health impacts. The project was funded by the Grand Challenges Canada (GCC) and South African Medical Research Council (SAMRC). The multidisciplinary approach encompassed aerobiological sampling using volumetric spore traps, meteorological data, a health survey via the standard International Study of Asthma and Allergies in Childhood (ISAAC) questionnaire (25), and Skin prick tests (SPTs) as a standard diagnostic test using pollen or fungi allergen extracts (26).

The structured mutivariate measurement parameters were used as independant variables to map the incidence of airborne pollen profiles allergen with focusing on asthma, AR, and eczema, seasonal differences, and exposure to allergenic aerospora in the Vaal Airshed Priority Area (VTAPA). VTAPA is an area in South Africa affected by air pollution at a globally significant level (27). Potchefstroom was selected for comparison as a nearby city in the same biome (Grassland with patches of savanna as described in Neumann et al. (23) and under similar climatic conditions but outside VTAPA.

This study is the first to report sensitization to grass, weed, and tree pollen species and fungal spores in Potchefstroom, South Africa. The results provide essential information about pollen and fungal types sensitization that can be applied to improve the diagnosis of allergic rhinitis and asthma in South Africa and review the testing aeroallergen panel of South African allergic working groups (SAARWG). In the present study, we aimed to identify and analyze the characteristics of a population with an incidence of allergic pollen species to establish a prototype of the regional incidence of seasonal pollen and associated respiratory diseases.

## Materials and methods

### Study site

The study was conducted in Potchefstroom (26˚ 42' 53” S; 27˚ 05' 49” E), a city in JB Marks Municipality, North-West Province (28). Potchefstroom is home to the largest campus of North-West University (NWU), which significantly influences the city's economy, culture, and social dynamics. The city covers an area of c. 55 km^2^ with a total of 201,000 residents (2024) (23).

The regional climate is characterized by summer rainfall with a mean average rainfall ranging between a maximum of >100 mm in December and January and <10 mm in June and August (29). The mean temperature fluctuates between c. 22°C in January, and >10°C -with common frost- in June/July (29).

**Figure 1:**
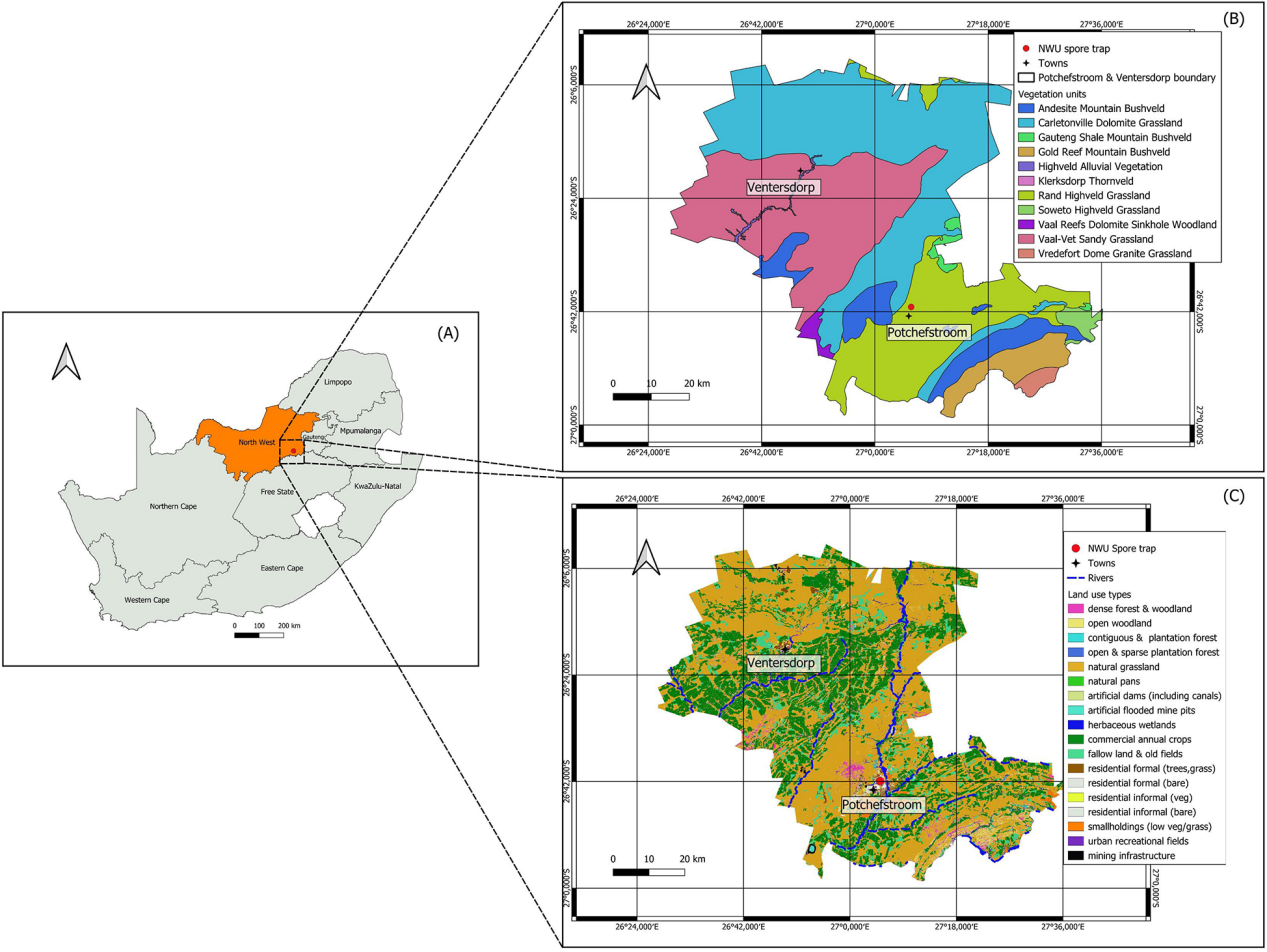
Geographical description of **(A)** the study area, **(B)** vegetation units, and **(C)** land use in the city of Potchefstroom.

Potchefstroom is situated along the Grassland Biome-Savanna Biome ecotone at the confluence of three vegetation units, namely the Rand Highveld Grassland, Carletonville Dolomite Grassland and Andesite Mountain Bushveld (10, 23) (Figure 1). These vegetation units include indigenous trees, such as *Searsia* spp. (karree tree), *Combretum* spp. (bushwillow), and African acacias (*Vachellia* spp., *Senegalia* spp.) (compare Neumann et al. (23).

All vegetation units have been altered and fragmented due to agriculture, forestry, urban development, and mining (Figure 1). Consequently, Potchefstroom hosts many exotic trees such as *Quercus* spp. (oaks), *Betula* spp. (birch), *Ulmus parvifolia* (Chinese elm), *Cupressus* spp./*Hesperocyparis* spp. (Cupressaceae, cypresses), *Platanus* spp. (plane trees), *Morus alba* (white mulberry)*, Morus nigra* (black mulberry), as well as weeds such as *Ambrosia* spp. (ragweed), and *Plantago* spp. (plantain) (23). Grasses include—next to indigenous grasses belonging to the genera *Eragrostis* (lovegrass)*, Panicum* (panicgrass)*, Cynodon* (Bermuda grass) – many exotic Poaceae such as *Pennisetum clandestinum* (Kikuyu grass) (23).

#### Aerobiological sampling

Aerospora monitoring, following SAPNET protocol, was conducted using a standard device 7-day Hirst-type volumetric spore trap (VPPS 2000 spore trap, Lanzoni s.r.l.), and set up at NWU, Potchefstroom campus, at a level of 12 m above ground in January 2023 (Figure 1) as part of the SAPNET monitoring initiative across South African cities (13). Aerospora monitoring was conducted continuously between January and December 2023, following the minimum requirements of the international requirement of aerobiology sampling (30). The sampler operates at a flow rate of 10 L/min, with the intake orifice aligned with the wind. Particles are collected on a glycerine jelly-coated plastic band around a drum, which moves at 2 mm/hour via a clockwork mechanism. Once a week, the adhesive tape is replaced, and the segments for each day are mounted on microscope slides and analyzed under a light microscope (×400). The monthly pollen index (pollen grains/m^3^) was calculated from the daily average airborne pollen concentrations for all major taxa contributing >1% of the annual pollen index. To compare the frequency of allergenic symptoms occurrence with aerospora temporal variation, we selected dominant pollen types based on our first insights into the pollen spectrum description of Potchefstroom (23). In addition, we included three fungal spores of the genera *Alternaria*, *Cladosporium* and *Aspergillus/Penicillium*, which were identified as dominant fungal spores in the North-West province of South Africa (31) and monthly spores indexes were calculated (fungal spores/m^3^). Due to technical issues with the sampler and data gaps in 2024, we chose to use the dataset from January 2023 to December 2023 to create a more comprehensive profile of the aerospora spectrum in Potchefstroom and evaluate the sensitization profile of recruited participants (compare Neumann et al. (23).

#### ISAAC questionnaires

To determine the prevalence of airborne allergens, with an emphasis on asthma, allergic rhinitis, and eczema, among individuals residing in the city of Potchefstroom, the symptoms questionnaire was adapted from the International Study of Asthma and Allergies in Childhoods (ISAAC) (25). Local allergologists and family physicians and, members of the research team, revised standardized ISAAC questionnaire, incorporating a few adjustments to the original questionnaire including the following enquiries: *Has a professional doctor confirmed your disease conditions? Which month of the year do you experience blocked nose, watery and itchy eyes?*

After receiving approval from NWU's Health Research Ethics Committee (HREC), informed consent and approval for participation in the study questionnaire and SPTs were obtained from the volunteers' participants from 21 to 26 October 2024. The sample comprises male and female students from different ethnicities living near or on NWU Potchefstroom campus. The collected information about adult symptoms was correlated with the average pattern of airborne pollen and fungal spore seasonality in Potchefstroom's atmosphere from 2022 to 2023.

#### Skin prick test

Before performing the procedure, a total of 202 volunteers' participants were screened for allergic rhinitis and/or asthma symptoms and for the intake of antihistamines and other medications that may inhibit the SPT results between. All volunteers' participants with antihistamine intake from seven days prior until the day of SPT were excluded. Participants with uncontrolled allergic rhinitis and/or asthma symptoms were exempted from SPTs. Pollen extracts purchased from Immunospec (Spain) includes allergens from Poaceae: *Cynodon dactylon* (Bermuda grass), *Lolium perenne* (ryegrass), *Zea mays* (maize), six mix grasses (*Poa pratensis* (Kentucky Blue), *Festuca pratensis* (meadow fescue), *Dactylis glomerata* (orchard grass), *Phleum pretense* (Timothy), *Holcus lanatus* (Yorkshire frog), Cupressaceae: *Hesperocyparis arizonica* (Arizona cypress), Fagaceae: *Quercus robur* (English oak), Platanaceae: *Platanus* sp. (plane tree), Ulmaceae: *Ulmus campestris* (field elm), Moraceae: *Morus alba* (white mulberry), Betulaceae: *Betula verrucosa* (silver birch), Oleaceae: *Olea* sp. (olive tree), weed mix (*Artemisia* sp. (wormwood), *Chenopodium* sp. (goosefoot), *Salsola* sp. (saltwort), *Plantago* sp. (plantain)), Asteraceae: *Ambrosia* sp. (ragweed), fungal spores including *Aspergillus fumigatus, Alternaria alternata, Penicillium notatum,* and *Cladosporium herbarum.* Due to lack of standardization and ambiguity common names for fungi are not provided here (compare (32).

SPTs were performed by three medical professionals with expertise in allergology and immunology on the participant's forearm with a standardized solution of allergens and no adverse events were reported. A mean wheal diameter of 3 mm or greater was used to indicate the presence of specific IgE in the allergen tested. Monosensitization was defined as a single sensitivity to one allergen and polysensitization was defined as a response to at least two or more allergens.

**Figure 2:**
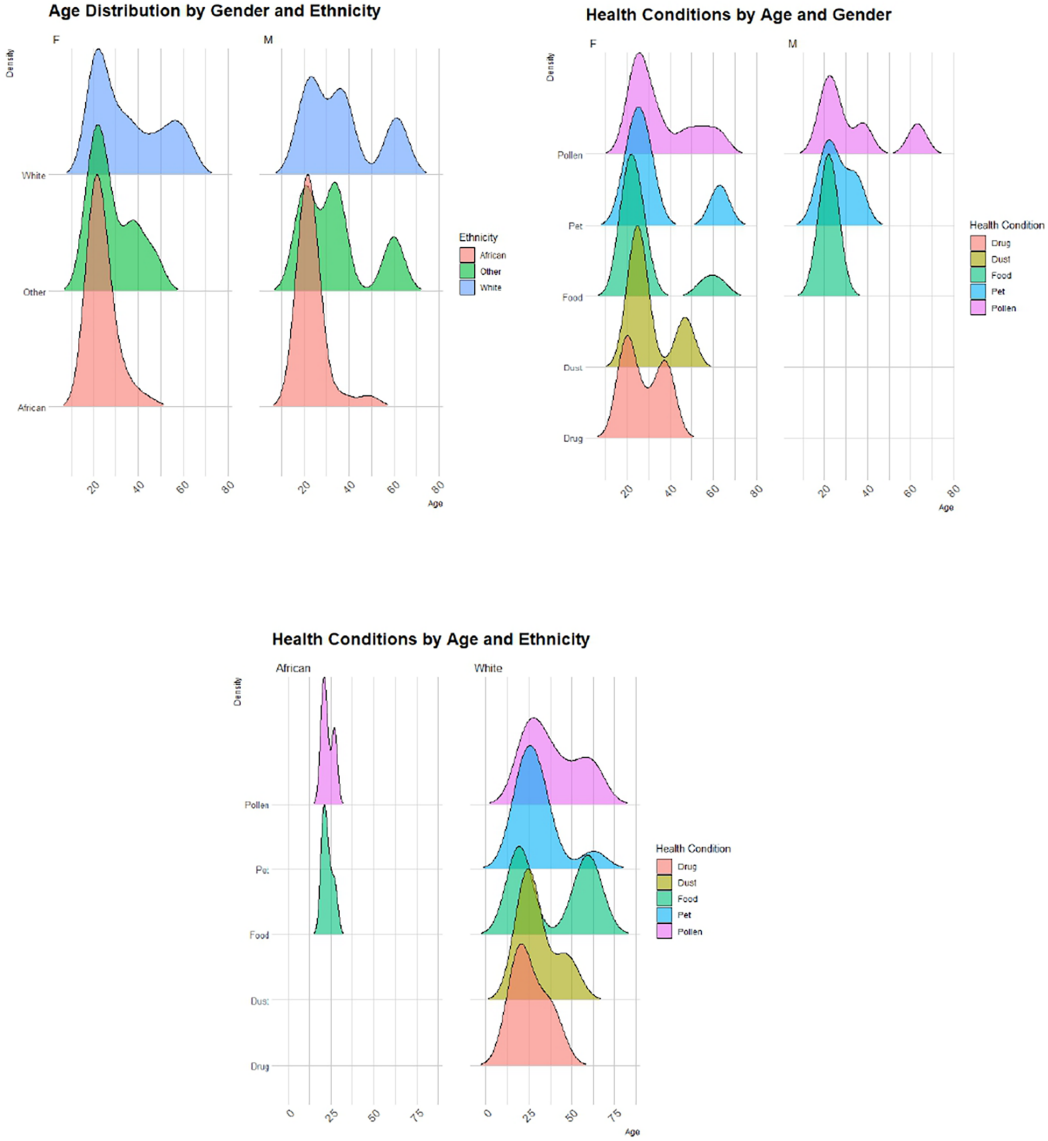
Demographic characteristics of patients with aeroallergen-positive SPT reactivity. *others: Colored and not specified.

The demographic variables and characterization of the participants with sensitivity (*n* = 184) are shown in Figure 2. Among the patients with sensitivity, 27% were male and 73% female, with an age range of 18–64 years and a mean of 29 years.

#### Statistical analysis

The data visualization and statistical analyses were performed using IBM Statistical Package for Social Sciences software SPSS (Version 29.0.2.0, released 2023, Armonk, NY: IBM Corp). Spearman's correlation explored the prevalence of symptoms and their relationship with aerospora particle levels from January to December 2023. Pearson's Chi-square tests examined associations between demographic characteristics (gender and ethnicity) and SPT results. Age parameters were excluded from the Chi-square analysis due to homogeneity of variance (highest tested participants with age group 18–25 years) linked to the characterization of the study locality on a university campus. Ethnicity was categorized as “African”, “White”, or “Other”, with smaller groups such as colored and not specified combined into “Other”. The SPT reactions were classified as positive or negative. Participants who did not exhibit a positive reaction to the positive control test were not considered in the Chi-Square tests. For all analyses, a *p*-value (*p* *<* *0.05*) was considered statistically significant.

## Results

### Aerobiological data

Table 1 illustrates the monthly variation in pollen counts for the dominant tree, grass, and weed taxa from January 2023 to December 2023. Grass pollen concentrations peaked in March (331 pollen grains/m^3^). *Ambrosia* (ragweed) pollen exhibited high concentrations during March (140 pollen grains/m^3^) and April (149 pollen grains/m^3^). Tree pollen, including that of Cupressaceae (cypresses), showed a notable monthly concentration peak in August (357 pollen grains/m^3^), followed by increased monthly values of *Platanus* (plane) (506 pollen grains/m^3^) and *Morus* (mulberry) (219 pollen grains/m^3^) in September. *Ulmus* (elm) pollen was highest in December (595 pollen grains/m^3^). In contrast to pollen, fungal spores showed a stable pattern throughout the year, with peaks occurring in March for *Cladosporium* (35,202 spores/m^3^) and *Alternaria* (2,387 spores/m^3^), and in July for *Penicillium* and *Aspergillus* (193 spores/m^3^).

**Table 1:** Monthly concentrations of common airborne pollen (pollen grains/m^3^) and fungal spores (fungal spores/m^3^) (January 2022-December 2023). Poa: Poaceae (grasses); Cup: Cupressaceae (cypresses); Quer: *Quercus* (oak); Plat: *Platanus* (plane); Ulm: *Ulmus* (elm); Bet: *Betula* (birch), Ole: *Olea* (olive); Art: *Artemisia* (sagebrush); Ama: *Amaranthus* (amaranth); Plan: *Plantago* (plantain); Mor: *Morus* (mulberry), Alt: *Alternaria*, Clad: *Cladosporium*; Peni: *Penicillium*; Asp: *Aspergillus*.

	Concentrations/month													Total/year
		Jan	Feb	Mar	Apr	May	Jun	Jul	Aug	Sep	Oct	Nov	Dec	
Pollen	Poa	134	133	331	84	17	6	6	3	1	2	11	89	817
	Cup	16	5	12	6	0	5	52	357	21	1	1	3	479
	Quer	26	2	22	4	3	3	1	2	41	4	0	0	108
	Plat	22	0	0	0	0	0	0	278	506	5	1	0	812
	Ulm	0	0	14	4	1	0	1	0	0	0	0	595	615
	Bet	0	0	0	0	1	0	0	7	34	5	1	1	49
	Ole	6	0	0	3	0	0	0	0	0	4	19	10	42
	Art	0	0	26	4	0	0	0	0	0	0	1	0	5
	Mor	4	1	3	0	0	1	0	2	219	2	1	1	234
	Ama	32	62	40	4	1	1	2	0	0	0	0	5	147
	Plan	77	7	19	3	1	1	0	0	1	2	33	59	203
	Amb	0	6	140	149	6	7	0	0	0	0	0	0	308
Fungal	Alt	423	449	2,387	962	701	276	186	64	24	45	71	15	5,603
	Clad	1496	1143	3,5202	8748	1,1322	4783	1214	341	88	436	1010	121	6,5904
	Peni/Asp	93	232	287	54	0	46	193	150	89	57	60	17	1,278

### Prescreening questionnaire response

The prevalence rates of asthma, AR, and eczema are shown in Supplementary Table S1. According to the ISAAC questionnaires that included self-reported symptoms, a total of 27 participants (13%) were confirmed to have asthma, 85 participants (42%) had allergic symptoms (e.g., skin itching, and runny nose) and 54 participants (27%) had urticaria. In assessing the severity of asthma, AR, and eczema symptoms, 29 (14%), 176 (87%), and 39 (19%) participants experienced wheezing attacks, sneezing, and eczematous skin rash, respectively, in the past 12 months. Regarding sleep disturbances, 11 participants (3%) were awake more than once a week due to wheezing attacks, and 12 participants (6%) experienced an itchy rash during one or more nights per week while sleeping over the past 12 months. Furthermore, 14 (7%) participants reported physician-diagnosed chronic respiratory diseases and 42 (21%) reported asthma.

### Overall of sensitized participants

**Figure 3:**
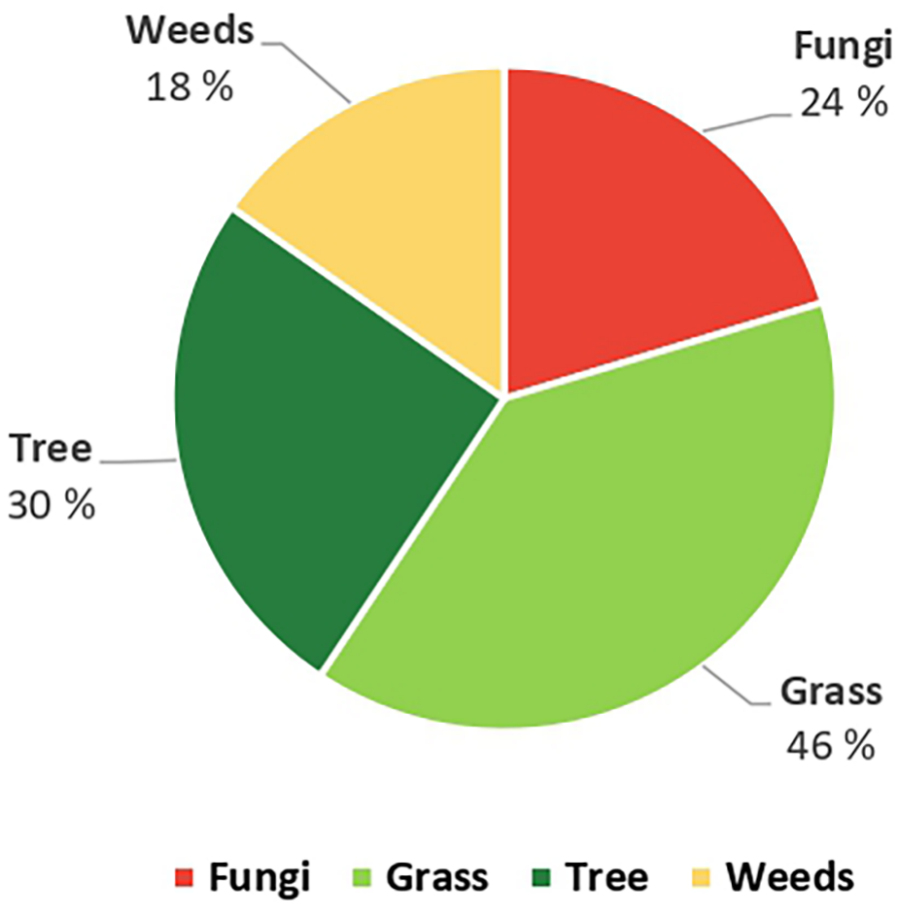
Total positive SPT reactions to pollen and fungal aeroallergens in Potchefstroom.

A total of 24% (45) of the participants had positive sensitization to fungi and 57% (104) to pollen; 19% (35) had positive reactions to both pollen and fungal spores. 19 participants had a positive SPT to one aeroallergen compared to 88 participants that assessed the prevalence to more than two aeroallergens. A total of 13 (12%) participants were monosensitized to one pollen allergen. While a total of 33 (73%) participants were monosensitized to one fungal spore type. Figure 3 shows the overall results of positive SPT reactions for all the aeroallergen panels tested. A significantly higher percentage of allergic reactions was observed for the grass pollen (46%) than for tree pollen (30%). In contrast, fewer participants reacted to fungi (24%).

**Figure 4:**
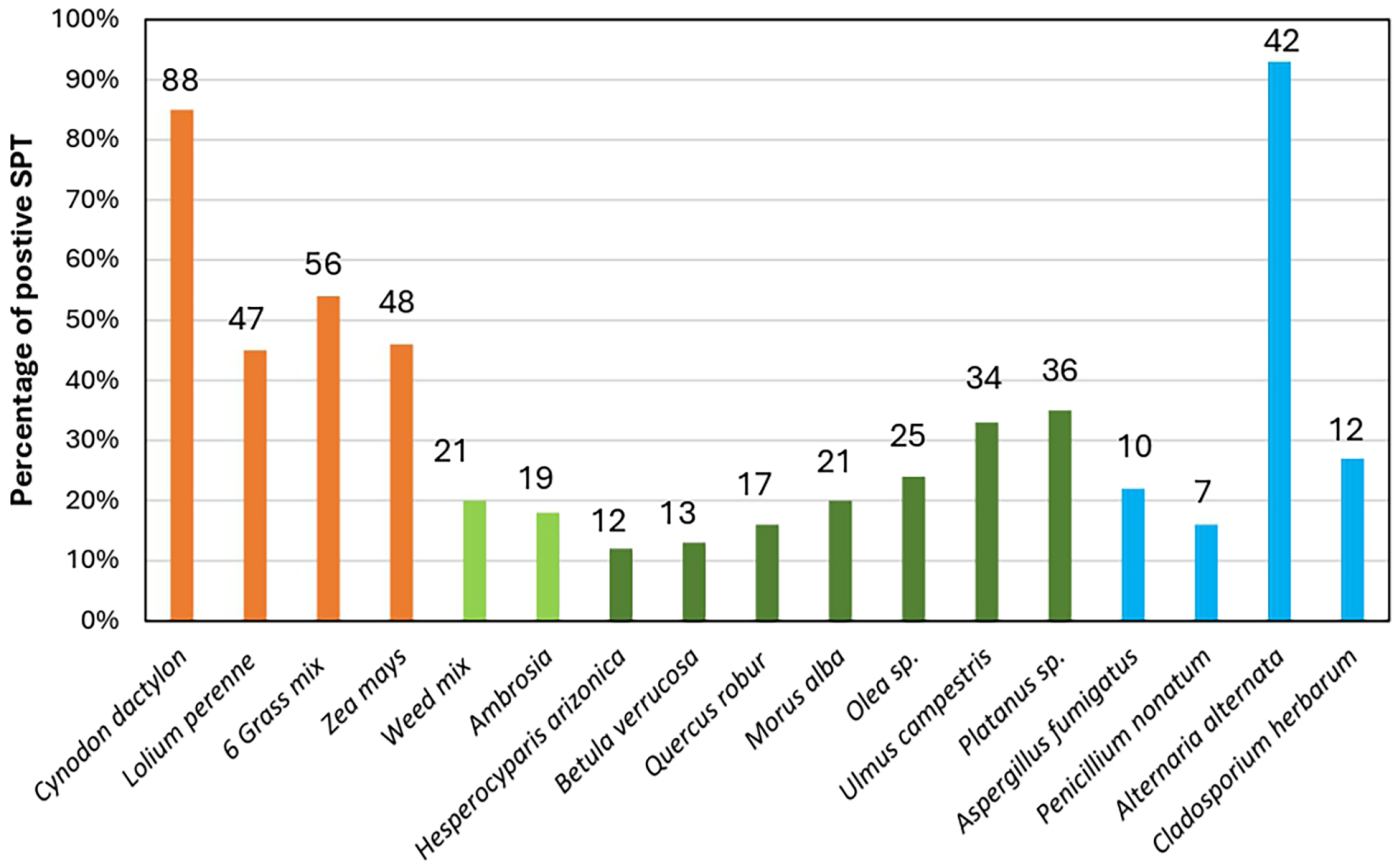
Percent (bars height) and frequency of positive sensitization rates (numbers in the top of bars) of participants in response to individual aeroallergens. *6 Grass mix: *Poa pratensis* (Kentucky bluegrass), *Festuca prantesis* (meadow fescue), *Dactylis glomereta*, (orchardgrass), *Phleum pratense* (Timothy grass), *Holcus lanatus* (Yorkshire fog), * Weed mix: (*Artimisia* (sagebrush), Chenopodium (goosefoot), Salsola (saltwort), Plantago (plantain).

Figure 4 illustrates the frequency of patients who tested positive for SPTs in response to tree, grass, and weed categories of allergen pollen extracts. Among the tree category, *Platanus* (plane) pollen allergen extract had the highest frequency rate of positive SPT results (36 patients, 35%), followed by *Ulmus* (elm) (34 patients, 33%) and *Olea* (olive) (25 patients, 24%). Among the grass pollen extracts category, *Cynodon dactylon* (Bermuda grass) allergen showed higher positive results (88 patients, 85%) compared to the allergen of *Zea mays* (maize) pollen, which had 48 patients (46%). Among participants who tested positive for pollen sensitivity, 21 (20%) reacted to the weed mix pollen: *Artemisia* (sagebrush), *Chenopodium* (goosefoot), *Salsola* (saltwort), and *Plantago* (plantain), whereas 19 (18%) exhibited sensitivity to *Ambrosia* (ragweed) pollen*.* Regarding fungal the spores category, the highest sensitivity frequency was observed to *Alternaria* (93%), followed by *Cladosporium* (27%).

### Relationship between symptoms and monthly aeroallergens concentrations

**Figure 5:**
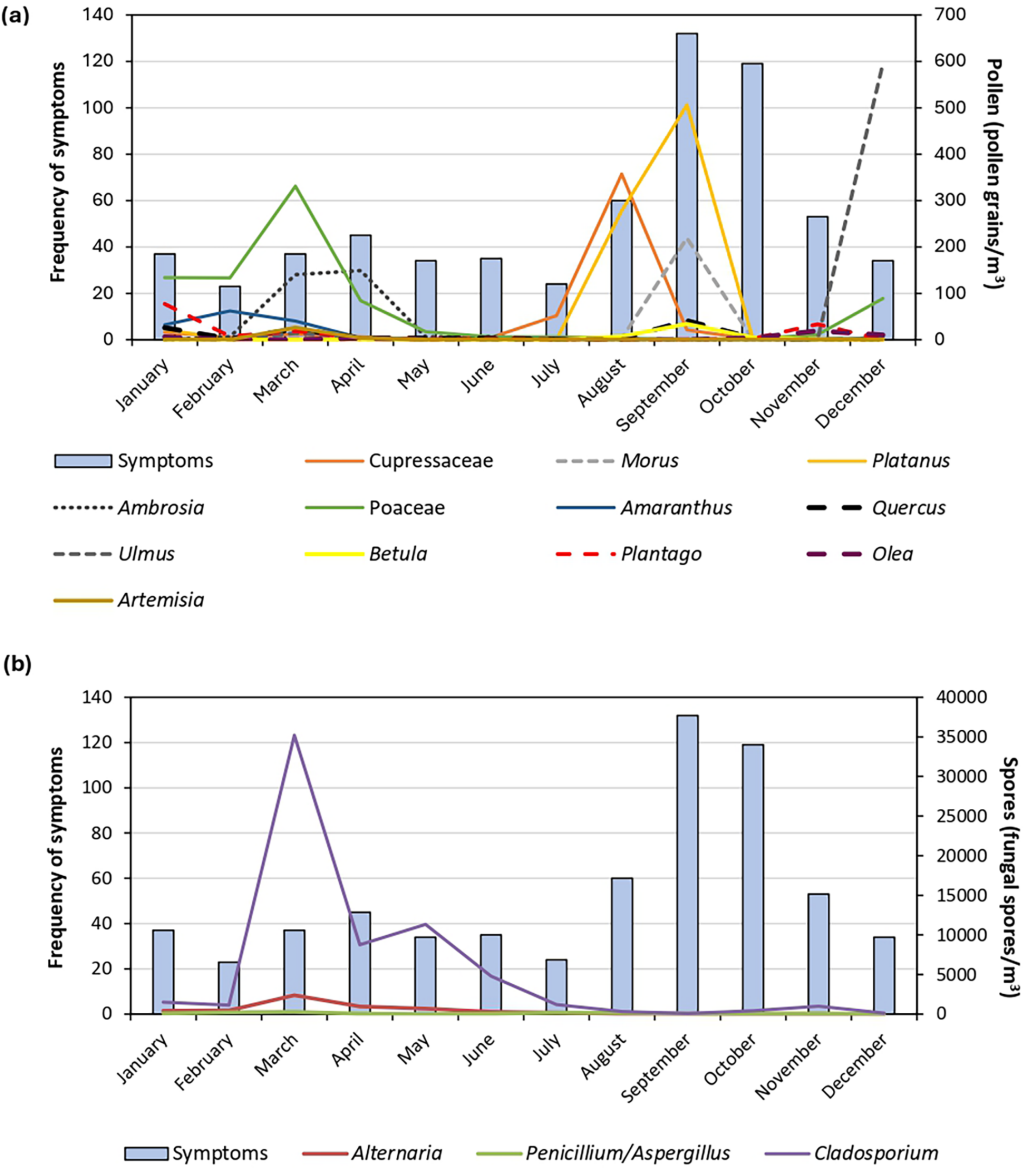
Association between the frequency of induced allergic symtomps and seasonal airborne **(a)** pollen and **(b)** fungal spores monthly concentrations. *Common names of taxa provided in text.

Figure 5 compares the monthly frequency of seasonal allergic rhinitis symptoms with the dynamics of airborne pollen and fungal spore counts. Following the dominant airborne pollen distribution described above (Table 1), almost all study participants reported allergic health conditions (blocked noses) throughout the year and exacerbation during the spring months (August, September, and October) in parallel with the increased monthly pollen concentrations of Cupressaceae, *Morus*, and *Platanus* and less frequent during summer months (January to April) with increased Poaceae and *Ambrosia* pollen loads (Figure 5). For fungal spores, there is no direct correlation between symptom conditions and the highest concentrations of *Cladosporium* fungal spores (Figure 5). Moreover, the statistical correlations between Cupressaceae, *Morus*, *Platanus*, Poaceae and *Ambrosia* pollen concentrations and actual symptoms reported by participants were analyzed (Supplementary Table S2).

To investigate the relationship between each individual aeroallergen and allergic rhinitis symptoms, Spearman's rank correlation coefficients were calculated (Table 2). Symptom prevalence was positively correlated with the following pollen concentrations: *Betula* (birch)*, Morus* (mulberry), *Platanus* (plane), and *Quercus* (oak).

**Table 2:** Spearman's correlation coefficient results for symptoms vs. aeroallergens concentrations.

Aeroallergen	*r*	*p*-value
*Alternaria*	−0.33	0.295
*Aspergillus/Penicillium*	−0.06	0.854
*Cladosporium*	−0.305	0.335
Poaceae	−0.531	0.076
*Zea mays*	−0.044	0.892
*Betula*	**0**.714**	0.009
Cupressaceae	0.102	0.753
*Morus*	**0**.505**	0.094
Olea	0.211	0.51
*Platanus*	**0**.814**	0.001
*Quercus*	0.496	0.101
*Ulmus*	−0.423	0.17
*Amaranthus*	**−0**.678*	0.015
*Ambrosia*	−0.286	0.368
*Artemisia*	0.166	0.607
*Plantago*	−0.066	0.839

The values in bold indicate a statistically significant relationship between aeroallergens and symptoms.

** Correlation is significant at the 0.01 level (2-tailed).

* Correlation is significant at the 0.05 level (2-tailed).

### SPTs vs. gender and ethnicity

Pearson's Chi-square test results suggest a significant association between gender and SPT results for the three allergens, namely *Platanus* (plane), *Olea* (olive) and *Betula* (birch). Furthermore, regarding the association between ethnicity and SPT results, two allergens showed a significant association at α=0.05: *Platanus* (plane tree) and Cupressaceae (cypresses). If a significance level of α=0.1 is chosen, two more allergens display significance, namely *Lolium* sp. (ryegrass) and the 6-grass mix,which are significantly associated with the SPT results and ethnicity. The results of the Chi-Square test are provided in Supplementary Table S3.

## Discussions

Generally, aerospora components are not universally distributed and do not possess the same allergenic impact on humans everywhere while climate change further exacerbates this variability (33). The occurrence of pollen allergies is influenced by local vegetation, the duration of allergen exposure due to different seasonalities, genetic predispositions to respiratory allergies, and other factors, such as increased urbanization, air pollution, and antibiotic usage (34). It is well-documented that inhalation of allergic pollen grains causes clinical symptoms of respiratory diseases (34). Evidence of high sensitization rates to Poaceae, especially *Cynodon dactylon* (Bermuda grass) have been documented in numerous countries such as Argentina (35), and Philippines (36), and African countries such as Madagascar (37), Zimbabwe (38), Uganda, Kenya, and Ethiopia (39), where the impact of climate change is expected to intensify grass pollen production and the burden of allergic diseases (15). In contrast, sensitization rates to other pollen types are less commonly reported across Africa, with the majority of reports coming from southern African countries (1).

The high cost of these standardized commercial extracts and allergy diagnosis tests discourages many individuals from being diagnosed in most African countries including, South Africa (40). In the case of the national reported prevalence rate, respiratory health metrics data were only available for patients with access to private healthcare and the data may not be representative of patients from disadvantaged backgrounds. SPT testing data could not be compared between all existing pollen or fungal spores aeroallergens and across all regions, as some SPT allergen profiles differ regionally. Furthermore, the imported pollen extracts do not always align with the local plants found in these regions, limiting their ability to precisely identify the specific sources of sensitization. As a result, these tests typically provide only a general overview of sensitization rather than an accurate local assessment. The diagnosis of sensitivity and specificity of standardized allergen extract mediated SPT testing are limited primarily to larger towns and cities in South Africa (41).

Recommendations regarding prevailing aeroallergens and a panel of IgEs were established in 2014 and were subsequently modified by the Allergic Rhinitis Diagnostic Working Group (ARDWG). In 2023, the ARDWG provided up-to-date testing recommendations on the panel of indoor/outdoor aeroallergens as the first-line appropriate diagnosis for South Africa, including house dust mites (HDM) (*Dermatophagoides pteronissinus* [Der p] and *farinae* [Der f]), cats, dogs, moulds (including *Alternaria*, and *Aspergillus*), and outdoor allergens (*Lolium* sp. (ryegrass), *Cynodon dactylon* (Bermuda grass), *Platanus* sp. (plane), and Cupressaceae (Cypresses)). ARDWG recommends that, considering that pollen data are not yet comprehensive for all areas across South Africa based on early pollen monitoring data (14) and the SAPNET website (https://pollencount.co.za), other allergenic pollens, such as those from trees and weeds, may be significant in specific regions, and a local common aeroallergen should be considered (41).

In this study, we analyzed the sensitization profile of adults living in Potchefstroom. A new emphasis on allergen evidence risk exposure was discussed and established with local health providers, following the first report of common pollen and fungal spore spectrum and abundance variation during a continuous aerobiological monitoring year January 2023–December 2023 (23). A screening of sensitization profiles to grass, trees, weeds, and fungi using standard aeroallergen extracts was conducted in subjects with self-reported allergic disease and asthma symptoms. In Potchefstroom, the positive sensitization pattern shows that grass accounted for the highest positive rate at 46%, followed by tree pollen (30%), fungal spores (24%), and weed pollen (18%).

Grasses are reported as major pollen allergens in many subtropical and temperate climate regions (42). In previous associated clinical research studies, grass pollen has been identified as a major aeroallergen responsible for upper airway allergic diseases in South Africa, with an early significant report by Ordman (17). South Africa has >967 indigenous Poaceae species and 115 naturalized species, including cultivated varieties such as *Pennisetum clandestinum* (Kikuyu grass), *Cynodon dactylon* (Bermuda grass), and *Lolium perenne* (ryegrass). The latter have been introduced with exception of indigenous *Cynodon dactylon* and are widespread, e.g., at the Cape Peninsula (43) and beyond. Visser et al. (44) mentioned at least 256 alien grass species of which 37 became invasive. A retrospective review of allergy testing data collected at AMPATH, private pathology provider in South Africa, covering the period from 1 January 2016 to 31 December 2017, analyzed SPT profiles from allergy clinics in four provinces. The findings revealed high sensitization rates to grass pollen (20). *Cynodon dactylon* (Bermuda grass) is a prominent allergen with positive sensitization rate of 30% in Western Cape, 31% in Gauteng, 29% in North West, 24% in Mpumalanga and Eastern Cape, while lower sensitization was recorded in KwaZulu Natal (15%) (20).

*Cynodon dactylon* (Bermuda grass) showed 84.6% of a positive reaction potentially favoring an increase in the allergy sensitization profile of tested participants, higher than obtained in other South African regions. It is essential to highlight that from a geographical point of view, Potchefstroom is located in a region where Grasslands and Savannas are the dominant biomes and grasses are ubiquitous (10). In Potchefstroom, the flowering periods of grasses are noteworthy [Table 1 and Figure 5 (23)]. Grass was the dominant pollen detected throughout the year, presenting a long pollen season, with the season peaking throughout summer (from December) until autumn (April) and a second peak in November (23). Because of the long grass flowering season, grass pollen allergy symptoms may be experienced for up to 10 months of the year (45), resulting in a high prevalence of persistent symptoms caused by pollen allergens (41).

Generally, grass pollen grains in Potchefstroom show a high daily mean concentration (23). The European Academy of Allergy and Clinical Immunology (EAACI) suggests that a grass pollen count of 50 pollen grains/m^3^ evokes allergic symptoms in individuals sensitized to pollen (46). Threshold values of 10 pollen grains/m^3^ and 12 pollen grains/m^3^ of grass pollen concentration were reported for emergency visits and hospital admissions occurrences (47).

Rooyen et al. (20) reports pollen from *Zea mays* (maize) as a significant allergen for individuals in areas where this species is cultivated, such as Gauteng (35%), North-West (25%), and Mpumalanga (19%). *Zea mays* pollen has been identified as a significant aeroallergen, with sensitization reported in 42.6% of atopic children, as noted by Mercer and van Niekerk (1991) using SPTs, 2% (48) in Free State using ImmunoCAP radioallergosorbent, and 56% in Bloemfontein (48) results of SPT to *Zea mays* (maize) pollen extract. A similar pattern was observed in our study; notably a high sensitization rate to *Zea mays* (maize) (46.2%). *Zea mays* pollen was not detected during pollen monitoring in Potchefstroom. While *Zea mays* pollen is large and heavy and consequently travels shorter distances than other grass pollen grains (49), its aerobiological dispersal remains a testable hypothesis (50).

*Zea mays* (var. *indurata* or *mays*) (white maize) is a vital field crop in South Africa, serving as the primary staple food for much of the population, especially among low-income households (51). Maize has been identified as an allergen for many patients who developed allergic respiratory symptoms after occupational exposure to *Zea mays* pollen and presented a history of grass pollen allergy (52). Cross-reactivity between *Zea mays* (maize) and *Cynodon dactylon* (Bermuda grass) pollen has been suggested due to their closer genetic relationship compared to *Lolium* sp. (rye grass pollen) (53, 54). This indicates that some patients might be primarily sensitized to maize pollen, making it a potentially significant allergen in the region. Van Rooyen et al. (20) highlight the need for a maize-specific IgE allergen component to identify such patients accurately.

Previous reports on outdoor allergens and environmental health exposure in South Africa, mainly in the Western Cape (55, 56), Free State (57) and Gauteng (20) confirm that hay fever and seasonal conjunctivitis associated with grass pollen are more prevalent than those linked to tree and weed pollen, in contrast to the patterns observed in Europe (58). Allergies to pollen of exotic trees such as *Quercus* (oak), *Platanus* (plane tree), Cupressaceae (cypress family), and *Olea* (olive) were identified for South Africa (45, 58) and are supported by aerobiological studies (14). *Olea* which includes both indigenous and exotic taxa- were causing most of the allergic reactions in alignment with the highly abundant pollen of those taxa in spore traps in Gauteng (Pretoria, Johannesburg) (14). *Olea europaea* L. subsp. *africana* [(Mill.) P.S.Green] (wild olive), *Olea capensis* (L.) (ironwood), *Olea exasperata* (Jacq.) (sand olive) are indigenous to South Africa (59). The latter two taxa are restricted to the Cape although *O. capensis* also grows in other South African provinces but not in North-West (59). The European olive, *Olea europaea* (L.) (common olive), is planted as a fruit tree at the Cape and as an ornamental tree throughout the subcontinent (23, 60).

South African urban green infrastructures, including in Potchefstroom, are characterized by the abundance of allergenic alien tree species introduced from the northern hemisphere as ornamental trees, including *Cupressus* spp. (cypresses), *Platanus* spp. (plane trees), *Pinus* spp. (pines), *Olea europaea* subsp. *europaea* (European olive), and *Quercus* spp. (oaks) (11, 23), and highly allergenic invasive ragweed, i.e., *Ambrosia artemisiifolia* (common ragweed) and *Ambrosia trifida* (giant ragweed) (24). Their abundance and marked allergenicity confirm the cause of pollinosis symptoms, illustrating an important issue in the South African environment.

Our study provides insights for northwestern South Africa and specifically for the Potchefstroom, by facilitating the first multi-centre research data collection. This includes accelerated recruitment of atopic population and the examination of tree and weed allergen positivity rates, previously unreported. A high prevalence of sensitization to *Platanus* (plane) (35%)*, Ulmus* (elm) (33%), *Olea* (olive) (24%) and *Morus* (mulberry) (20%) pollen extract, in addition to *Ambrosia* (ragweed) (18%) was noted. This is in good agreement in respect to pollens of *Platanus* (plane), *Olea* (olive)*,* and *Morus* (mulberry), in comparison to aerobiological data from other sites in the Grassland and the savanna, such as Kimberley, Bloemfontein, Johannesburg and Pretoria (14). A possible explanation for this observation in Potchefstroom is the strong correlation between pollen load and land cover taxa distribution in Potchefstroom. Potchefstroom is characterized by neophytic trees such as *Platanus acerifolia* (London plane), *Morus alba* (white mulberry), *Ulmus parvifolia* (Chinese elm), and *Ulmus americana* (American elm) which are found along roadsides, in parks, and in gardens (23).

The contribution of fungal spores to allergy symptom profiles among residents was observed, with a high SPT prevalence for *Alternaria* (93%) and *Cladosporium* (27%) and a high occurrence of *Alternaria* and *Cladosporium* spores especially in March-May (Figure 5). *Alternaria* is recognized globally as one of the main fungal aeroallergens (61). Earlier allergy studies in South Africa, based on SPT data provided by a laboratory, identified *Alternaria* as predominant fungal sensitizer. These studies were conducted in Cape Town (55) and the Free State (62). Our findings support previous tests on the effect on lung function of fungal spores amongst school children in the Western Cape, where *Alternaria* and *Cladosporium* were isolated as severe allergens, but fungal spores were only monitored during summer and winter (63, 64).

The allergenicity of most fungal spores remains poorly understood, although their high presence in aerobiological monitoring (65) shows a weak correlation with sensitization patterns (66, 67). Notably, when fungal spores from aerobiological surveys are compared with SPT or specific IgE test results, their rankings may not always align. Although daily fungal spore levels did not exceed the low category on the Burge scale defining the allergy risk levels based on aerospora concentrations (68), these spores may still exhibit allergenic potential.

The statistical correlation analysis conducted between allergic symptoms and various monthly concentrations of airborne pollen types revealed a significant association between *Betula* (birch), *Morus* (mulberry), *Platanus* (plane), and *Quercus* (oak) pollen and symptoms among the participants of the current study (Supplement Table S3). The scarcity of correlations between the other pollen types and symptoms reported may be attributed to low pollen concentrations in the region or to threshold levels required to trigger associated symptoms (69, 70). Becker et al. (47) highlight that population sensitivity to pollen is influenced by local vegetation. They concluded that, when a plant species is prevalent in a particular geographical area, the population tends to tolerate higher concentrations of its pollen before experiencing symptoms. Several potential confounders could affect the relationship between pollen exposure and allergy, including weather, climate, environmental conditions, and human activities, which have a significant impact on the amount and diversity of pollen (71, 72). Factors linked positively or negatively to pollen exposure might lead to misleading associations. Air pollution, more common in industrial and highly polluted areas, has been shown to potentially enhance the effects of certain aeroallergens, thereby increasing the prevalence or severity of allergic rhinitis and asthma (73, 74). It appears unlikely that high pollen exposure directly raises the risk of developing allergic rhinoconjunctivitis or asthma, although it can provoke symptoms in susceptible individuals (75). The associations between atmospheric pollen load and allergy-triggered symptoms observed in this study were weak (Supplement Table S2). This suggests that pollen exposure may have little to no influence on the prevalence of atopic diseases in the residents of Potchefstroom.

ISAAC also screens for allergic rhinitis, and it is possible that patients may be sensitized to other common aeroallergens, such as house dust mites, insects (e.g., cockroaches), and pets (e.g., cats and dogs). In cases of local allergic rhinitis, patients may still have a true allergy, even if their allergy tests return negative results (76).

Moreover, since pollen counts fluctuate annually, relying on data from a single year may diminish the analysis's sensitivity. To create robust pollen calendars, a five-year cycle is recommended. Careful thought should also be given to the sensitization among races groups and gender.

The negative relation between adjustment for the self allergy symptom severity report obtained through the ISAAC questionnaire vs. monthly aeroallergen pollen load vs. sensitization is best explained by psychological factors of the participants overrating the intensity of their symptoms. Most participants did not have any previous knowledge about allergenic rhinitis and related symptoms. Similar studies found that reported symptom severity of allergic rhinitis did not correlate with weal size for any of the aeroallergens tested or with the number of positive responses on SPT but rather to psychological factors of hypochondriasis and somatic awareness (76).

A significant association between gender (male vs. female) and sensitization to aeroallergens, particularly a higher sensitization for the exotic tree pollen *Platanus*, *Olea*, and *Betula* among females. Similar findings have been reported in a previous study linked to the same project conducted in Vanderbijlpark, a polluted and highly instrustalized area within Gauteng province, with higher positive rates of pollen sensitization among females compared to males (77). Further, Jensen-Jarolim & Untersmayr (78), support the gender-associated sensitization evidence, through the key factors of mechanistic involvement of sex hormones in immune responses. Additionally, a significant association was observed between *Platanus*, *Cupressaceae*, *Lolium*, and a six-grass mix with ethnicity.

Regarding allergen-specific susceptibility related to ethnicity, evidence of racial differences in allergic sensitization among adults living in Vanderbijlpark was observed (77). Similarly, Wegienka et al. (79) highlighted racial differences in allergic sensitization among various racial groups. However, the underlying sources of these disparities remain unclear but might be influenced by factors such as genetics, socioeconomic status (e.g., income, education), and environmental conditions (e.g., particulate matter in quantity and type). Further research is needed to investigate combinations of factors including allergen-specific susceptibility vs. race or gender host susceptibility.

This study is limited by: a. short aerobiological dataset; b. this is the first population based study to evaluate the aeroallergen exposure in Potchefstroom; all participants were recruited from a university setting, which may not fully represent the demographic and/or socio-economic composition or occupational exposure patterns of the general population in this city. c. Skin prick testing is a specialized skill that requires both time and trained personnel to carry out effectively. Because of this, the number of SPTs tests we were able to perform was limited. In total, we could only complete 202 tests due to the time-intensive nature of the procedure and the scarcity of skilled personnel required to administer it (one doctor per day).

A first case study is reported here as part of an ongoing pilot research project that will include, in a later stage, individuals from the general public in terms of age distribution and socio-economic background. As a result, the findings should be interpreted with caution when considering their applicability to the wider community. Future studies should aim to include a more diverse sample to enhance generalizability; d. SPTs were conducted for the first time on participants without any previous knowledge on aeroallergen diversity and exposure health issues, e. as a consequence of d., participants frequently provide inaccurate self-reports on the monthly frequency of associated symptoms throughout the year.

## Conclusions & outlook

• This research study represent the first attempt of SPT series conducted in Potchefstroom, a small, rapidly growing city in the North-West province. These tests highlighted the significant role of allergens from exotic trees and weeds.
• Poaceae allergens, such as those from *Cynodon dactylon* (indigenous Bermuda grass), were found to be particularly important, with clear evidence of sensitization to grasses. To assess the alignment between SPT results and aerobiological data, it is important to note that, from a morphological perspective, differentiating Poaceae pollen under light microscopy is limited and requires improvement, such as through the integration of genetic studies (DNA barcoding) (80). This will enhance our understanding of grass pollen allergenicity.
• For the first time, sensitization to *Ambrosia* sp. (ragweed from North America) was documented for South Africa using SPTs. This taxon, present with several species in South Africa, is currently spreading and needs to be closely monitored and further investigated regarding its allergic potential (24).
• Additionally, the prevalence of fungal spores, most importantly *Cladosporium*, emerged as highly significant. DNA studies might enhance species-level knowledge in urban environments (81).
• The study underscored the need for local monitoring of certain allergenic pollen types, such as *Zea mays* (maize), which are currently underrepresented in aerobiological studies due to maize pollen grains being weakly reflected in spore traps**.** Spore traps lower to the ground, e.g., at 2 m height, might address this crucial lack of information.
• We suggest making SPTs, which are often not affordable for many South Africans, budget-friendly and promote SPTs being conducted in other regions in the country and across Africa.

## Data Availability

The raw data supporting the conclusions of this article will be made available by the authors, without undue reservation.
