# Supplementary material for: Exposure to outdoor aerospora and associated respiratory health risks among adults in Potchefstroom, North-West province, South Africa
Source: Front Allergy. 2025 Apr 15;6:1568669. doi: 10.3389/falgy.2025.1568669 (PMC12037600; doi:10.3389/falgy.2025.1568669)
Supplement: Supplementary file 1 [file Table1.docx]

Supplementary Material

**Supplementary Tables**

**Table S1.** Prevalence of asthma, allergic rhinitis, eczema, and associated symptoms in the study group (n = 202).

| Symptoms | N (%) |
| --- | --- |
| 1. Chronic respiratory diseases confirmed by a medical doctor | 14 (6.93) |
| 2. Allergic symptoms (skin itching, runny nose, watery eyes, etc?) for at least 3 days/nights lasting 3 months | 85 (42.08) |
| 3. Asthma | 27 (13.40) |
| 4. Poor lung function | 5 (2.47) |
| 5. Anaphylaxis/severe allergic reaction  During the previous 30 days | 5 (2.47) |
| 6. Dermatographism | 18 (8.91) |
| 7. Urticaria | 54 (26.73) |
| 8. Atopic dermatitis | 33 (16.33) |
| Asthma (during last 12 months)  1. Wheezing or whistling in the chest at any time in the last 12 months | 29 (14.35) |
| 2. Frequency of wheezing attacks in the last12 months  1-3 times  4-12 times  More than 12 | 11 (5.44)  7 (3.46)  4 (1.98) |
| 3. Frequency of wheezing attacks during sleeping in the last 12 months  Never woken with wheezing  Less than one night per week  One or more nights per week | 8 (3.96)  7 (3.46)  11 (5.44) |
| 4. Diagnosed with asthma during the last 12 months? | 24 (11.88) |
| 5. Chest sounded wheezy during or after exercise | 23 (11.38) |
| 6. Dry cough at night not associated with a cold or chest infection | 26 (12.87) |
| Allergic rhinitis (during last 12 months)  1. Sneezing, runny or blocked nose not associated with cold or flu | 176(87.12) |
| 2. Nose problem accompanied by itchy-watery eyes | 122(60.39) |
| 3. Hay fever | 152(75.24) |
| Eczema (during last 12 months) |  |
| Confirmed Eczema | 42 (20.79) |
| 1. Itchy rash during the last 12 months | 39 (19.30) |
| 2. Has this itchy rash at any time affected any of the following places  The folds of the elbows, behind the knees  In front of the ankles, under the buttocks  Around the neck, ears, or eyes | 8 (3.96)  2 (0.99)  12 (5.94) |
| 3. Has the rash cleared completely at any time during the last 12 months? | 23 (11.86) |
| 4. Frequency of itchy rash during sleeping in the last 12 months  Never  Less than one night per week  One or more nights per week | 16 (7.92)  11 (5.45  12 (5.94) |

**Table S2.** Lower triangle of correlation matrix: Symptom prevalence and pollen concentrations

**Table S3.** Pearson’s Chi-Square tests: Association between allergens and gender and ethnicity

|  | **Gender** | | | | **Ethnicity** | | | | |
| --- | --- | --- | --- | --- | --- | --- | --- | --- | --- |
| **Allergen** | $\chi^{2}(1)$ | $p$ | % F | % M | $\chi^{2}(1)$ | $p$ | % AF | % W | % Other |
| *Aspergillus* | 0.13 | 1 | 3.00% | 2.00% | 0.37 | 0.831 | 3.00% | 3.00% | 0.00% |
| *Penicillium* | 0.751 | 1 | 1.50% | 0.00% | 3.463 | 0.177 | 0.00% | 3.00% | 0.00% |
| *Alternaria* | 1.86 | 0.209 | 17.40% | 26.50% | 1.843 | 0.398 | 18.80% | 24.20% | 8.30% |
| *Cladosporium* | 0.123 | 0.663 | 3.00% | 4.10% | 2.498 | 0.287 | 2.00% | 6.10% | 0.00% |
| Bermuda grass | 0.13 | 0.739 | 43.90% | 46.90% | 2.295 | 0.317 | 49.50% | 37.90% | 50.00% |
| Ryegrass | 0.062 | 0.844 | 22.70% | 24.50% | 5.051 | **0.08*** | 21.80% | 21.20% | 50.00% |
| 6 Grass mix | 0.95 | 0.36 | 27.30% | 34.70% | 5.259 | **0.072*** | 28.70% | 25.80% | 58.30% |
| *Platanus* | 8.471 | **0.005**** | 13.60% | 32.70% | 6.336 | **0.042**** | 13.90% | 22.70% | 41.70% |
| *Cypress* | 0.896 | 0.463 | 4.50% | 8.20% | 6.82 | **0.033**** | 2.00% | 9.10% | 16.70% |
| *Quercus* | 0.642 | 0.404 | 8.30% | 12.20% | 2.108 | 0.348 | 6.90% | 13.60% | 8.30% |
| *Morus* | 2.998 | 0.115 | 9.10% | 18.40% | 1.804 | 0.406 | 8.90% | 15.20% | 16.70% |
| Ambrosia | 0.218 | 0.597 | 9.80% | 12.20% | 1.871 | 0.392 | 7.90% | 13.60% | 16.70% |
| *Zea maize* | 0.1 | 0.847 | 24.20% | 26.50% | 4.063 | 0.131 | 29.70% | 16.70% | 33.30% |
| *Ulmus* | 1.05 | 0.38 | 15.90% | 22.40% | 1.544 | 0.462 | 14.90% | 21.20% | 25.00% |
| *Olea* | 4.933 | **0.046**** | 9.80% | 22.40% | 3.231 | 0.199 | 8.90% | 18.20% | 16.70% |
| *Betula* | 5.086 | **0.046**** | 4.50% | 14.30% | 1.847 | 0.397 | 5.90% | 7.60% | 16.70% |
| Weed mix | 1.903 | 0.186 | 9.10% | 16.30% | 1.289 | 0.525 | 8.90% | 13.60% | 16.70% |
| ** : significant at $\alpha=0.05$  * : significant at $\alpha=0.1$  % F: percentage of female participants who had a significant SPT reaction to the allergen  % M: percentage of male participants who had a significant SPT reaction to the allergen  % AF: percentage of African participants who had a significant SPT reaction to the allergen  % W: percentage of White participants who had a significant SPT reaction to the allergen  % Other: percentage of Other participants who had a significant SPT reaction to the allergen | | | | | | | | | |

Note: It is important to acknowledge that the number and distribution of participants across the Gender and Ethnicity demographic groups may not be adequate for generalizing the findings to the broader study population.
